# Antiinflammatory, Analgesic and Antipyretic Activities of *Mimusops elengi* Linn

**DOI:** 10.4103/0250-474X.73908

**Published:** 2010

**Authors:** A. Purnima, B. C. Koti, A. H. M. Thippeswamy, M. S. Jaji, A. H. M. Vishwantha Swamy, Y. V. Kurhe, A. Jaffar Sadiq

**Affiliations:** Department of Pharmacology, KLES’s College of Pharmacy, Rajaji Nagar, Bangalore-560 010, India; 1 Department of Pharmacology, KLES’s College of Pharmacy, Hubli-580 031, India

**Keywords:** *Mimusops elengi*, antiinflammatory, analgesic, antipyretic, carrageenan

## Abstract

In the present study, 70% ethanol extract of *Mimusops elengi* Linn. bark was assessed for antiinflammatory, analgesic and antipyretic activities in animals. The antiinflammatory activity of ethanol extract of *Mimusops elengi* (200 mg/kg, p.o) was evaluated using carrageenan-induced paw edema and cotton pellet-induced granuloma models. Analgesic effect was evaluated using acetic acid-induced writhing and Eddy’s hot plate models and antipyretic activity was assessed by Brewer’s yeast-induced pyrexia in rats. The ethanol extract of *Mimusops elengi* (200 mg/kg, p.o) significantly inhibited the carrageenan-induced paw oedema at 3rd and 4th h and in cotton pellet model it reduced the transudative weight and little extent of granuloma weight. In analgesic models the ethanol extract of *Mimusops elengi* decreases the acetic acid-induced writhing and it also reduces the rectal temperature in Brewer’s yeast induced pyrexia. However, *Mimusops elengi* did not increase the latency time in the hot plate test. These results show that ethanol extract of *Mimusops elengi* has an antiinflammatory, analgesic and antipyretic activity.

Inflammation is a body defense reaction to eliminate or limit the spread of an injurious agent and is characterized by five cardinal signs, redness (*rubor*), swelling (*tumor*), heat (*calor*), pain (*dolor*) and loss of function (*function laesa*). The inflammatory process involves a cascade of events elicited by numerous stimuli that include infectious agents, ischemia, antigen-antibody interaction and thermal or physical injury[1,2]. Non-steroidal antiinflammatory drugs (NSAIDs) are widely used in the treatment of acute and chronic inflammation, pain and fever. But the greatest disadvantage in presently available synthetic drugs is that they cause gastrointestinal irritation and reappearance of symptoms after discontinuation. Therefore, there is a dire need for screening and development of novel, but better antiinflammatory drugs and indigenous medicinal plants could be a logical source to find these.

*Mimusops elengi* L. (sapotaceae) locally known as *Bakul*, *Pagademara* is a small to large tree found all over India. It is also cultivated in garden as an ornamental tree. It has been used in the indigenous system of medicine for the treatment of various ailments such as, dental disease, burning sensation, uterine disorders, ulcers, cardiac diseases, fever and the plant has been used as diuretic, astringent and aphrodisiac[3,4]. This plant has also been reported for antiulcer[5], antihyperlipidemic[6] and anthelmintic activities[7]. Phytochemical evaluation showed the presence of alkaloids, tannins, ursolic acid, steroids, querrcitol, lupeol and mixtures of triterpenoid saponins[8]. Recently it has been reported that traditional plants that contain saponins such as, *Panax ginseng*[9], *Alchornea cardifolia*[10] and *Bupleurum rotundifolium*[11] possessed antiinflammatory, analgesic and antipyretic activities. However there have been no published reports on the antiinflammatory, analgesic and antipyretic activities of *Mimusops elengi*. Thus the present study was undertaken to investigate the antiinflammatory, analgesic and antipyretic activities of alcohol extract of *Mimusops elengi*.

## MATERIALS AND METHODS

The bark of *Mimusops elengi* (ME) was collected from mature trees grown locally. The bark of the plant was authenticated in the Department of Botany, Shri Kadasiddheshwar Arts College and H. S. Kothambri Science Institute, Hubli. Experiments were carried out on Wistar rats weighing 150-200 g and mice weighing 20-30 g. Wistar rats were procured from National Institute of Mental Health and Neuro Sciences (NIMHANS), Bangalore and mice from KLES College of Pharmacy, Hubli, Karnataka. Animals were housed in standard polypropylene cages for one week to acclimatize to laboratory conditions before starting the experiment at a temperature of 24±2° and relative humidity of 30-70%. A 12:12 light:dark cycle was followed. They were given free access to standard rat feed and water *ad libitum*, but 12 h prior to an experiment; the rats were deprived of food but not water. The animal study protocols were approved by the institutional animal ethical committee.

The following chemicals procured from sources indicated in parentheses were used in this investigation, ethanol and CMC sodium (Nice Chemicals, Bangalore), carrageenan and anesthetic ether (Sigma Chemicals, Mumbai), diclofenac (SGS Pharmaceuticals, Pvt. Ltd., Delhi), acetic acid (S. D. Fine Chemicals, Mumbai), pentazocine (Ranbaxy Lab Ltd, Mumbai).

### Extraction and preparation of sample:

The bark was shade-dried, until free from moisture. Then, they were subjected to size reduction to get coarse powder of desired particle size. The powdered material was subjected to successive extraction in a Soxhlet apparatus using solvent petroleum ether (40-60°) and alcohol. Temperature was maintained on an electric heating mantel with a thermostat control. The extract was then concentrated to 3/4 ^th^ of its original volume using a rotavap apparatus. The concentrated extract was then transferred to a china dish and evaporated on a thermostat-controlled water bath till it formed a thick paste. This thick mass was vacuum dried in a desicator till it is free from moisture. The extract was subjected to chemical tests for the detection of phytoconstituents. Alcohol extract of ME was administered orally as suspension by triturating with 5% Tween 80.

### Acute toxicity studies:

Acute toxicity tests were performed on mice of either sex weighing between 20-30 g following OECD guidelines[12] after obtaining approval from the Institutional Animal Ethical Committee of KLESCOPH bearing No.: KLESCOPH/IAEC. Clear/2007-08.

### Carrageenan-induced paw oedema:

Rats were divided into three groups of six each. The first group served as control and received normal saline (0.1ml/ 10 g p.o.). The second group was administered diclofenac sodium (10 mg/kg p.o.) as the standard drug. The third group received alcoholic extract of ME (200 mg/kg p.o.). Oedema was induced by injecting 0.1 ml of 1% carrageenan suspension into the subplantar region of the right hind paw of the rats of all three groups 30 min later. The paw volume of animals was measured by means of a volume displacement technique using a plethysmometer before and 0.5, 1, 2, 3, 4 and 5 h after administering carrageenan[13]. Results were expressed as percentage of inhibition of oedema, calculated according to the following formula, percent inhibition = 100×(Vc–Vt)/Vc), where Vc is the volume of the oedema in control, Vt is the volume of oedema in animals treated with test extracts.

### Cotton pellet-induced granuloma:

Two autoclaved cotton pellets weighing 10±1 mg were implanted subcutaneously into both sides of the groin region of each rat[14]. The animals were divided into three groups containing 6 animals in each group. First group served as control and received normal saline daily at a dose of 0.1 ml/10 g p.o., second group received diclofenac sodium (standard drug) daily at a dose of 10 mg/kg p.o and third group received ME at a dose of 200 mg/kg p.o. daily for 7 consecutive days. After 7 days the animals were sacrificed by large doses of anaesthetic ether and the pellets together with the granuloma tissues were carefully removed, dried in an oven at 60^0^ weighed and compared with control.

### Writhing in mice:

The mice were divided into three groups of six each. The first group served as control and received normal saline (0.1 ml/ 10 g p.o.). The second group was administered diclofenac sodium (10 mg/kg p.o.) as the standard drug and third group received alcoholic extract of ME (200 mg/kg p.o.). Thirty minutes later, each mouse was given 0.1 ml/10 g i.p. of 1% acetic acid[15]. Five minutes after acetic acid induction, the number of writhes like abdominal muscle contraction, stretching of the hind limbs and trunk twisting were counted for 15 min. The results were expressed in percentage of inhibition.

### Tail clip method:

The mice were divided into three groups of six each. The first group served as control and received normal saline (0.1ml/ 10 g p.o.). The second group was administered pentazocine (10 mg/kg s.c.) as the standard drug. The third group received alcoholic extract of ME (200 mg/kg p.o.). The test was done by applying a metal artery clip at the base of the tail with its jaw sheathed with thin rubber tubing[16]. Those mice which did not show any effort to dislodge the clip within 15 s were rejected. The tail clip was applied 30, 60, 90 and 120 min after oral administration of extract. It was considered as a response if there was no attempt by the mouse to dislodge the clip within 15 s.

### Eddy’s hot plate method:

Mice were divided into three groups of six each. The first group served as control and received normal saline (0.1ml/ 10 g p.o.). The second group was administered pentazocine (10 mg/kg s.c.) as the standard drug and third group received alcoholic extract of ME (200 mg/kg p.o.). The test was carried out using Eddy’s hot plate apparatus[17], the temperature was set at 55±1°. Mice were placed on hot plate and recorded the reaction time in second for licking of hind paw or jumping with cut off time of 15 s. The reaction time following the administration of the test extracts, reference standard drug, and control saline vehicle were measured at 0, 30, 60, 90 and 120 min.

### Brewer’s yeast-induced fever:

Initial rectal temperatures were recorded using digital thermometer. Pyrexia was induced in animals by injecting 20 mg/kg (1ml/100 g) of 20% suspension of brewer’s yeast subcutaneously[18]. After 18 h, animals that showed an increase of 0.3–0.5^0^ in rectal temperature were selected. The alcoholic extract of ME (200 mg/kg), paracetamol (150 mg/kg) as standard drug and control saline vehicle (0.1ml/ 100 gm p.o.) were administered orally and rectal temperature was determined by digital thermometer 30 min before and 0.5, 1, 2, 3, 4, 5 and 6 h after extract or drug administration.

### Effect on body temperature:

The rats were divided into three groups of six rats. The first group served as control and received normal saline (0.1ml/ 100 g p.o.). The second group was administered paracetamol (150 mg/kg p.o.) as the standard drug. The third group received alcoholic extract (200 mg/kg p.o.). The test was conducted at room temperature of 28±1°. The rectal temperature of both the groups was recorded every 30 min for 4 h.

### Statistical analysis:

The results were expressed as the mean ± SEM. The results obtained from the present study were analyzed using one way ANOVA followed by Dunnett’s multiple comparison tests. Data was computed for statistical analysis by using Graph pad prism software.

## RESULTS AND DISCUSSION

Preliminary phytochemical studies showed the presence of carbohydrates, saponins, triterpenoids, tannins, flavonoids and glycosides in alcoholic extract of ME. The maximum nonlethal dose was found to be 2000 mg/kg; hence 1/10th of the dose was taken as effective dose (200 mg/kg). In acute inflammation model, the alcohol extract of ME at a dose of 200 mg/kg showed maximum inhibition of carrageenan-induced paw oedema at 3 ^rd^ h (30.9%) with a mean paw edema volume of 0.94±0.05 ml and diclofenac showed 36.8 % inhibition (0.86±0.027 ml) of oedema at 3 ^rd^ h when compared to control 1.36±0.014 (Table 1).

**TABLE 1:** EFFECT OF ALCOHOLIC EXTRACT OF MIMUSOPS ELENGI ON CARRAGEENAN INDUCED PAW OEDEMA IN RATS

Treatment	Rat hind paw volume (ml) (Percent inhibition)					
	30 min	1 h	2 h	3 h	4 h	5 h
Normal saline	0.91±0.20	0.95±0.02	1.07±0.03	1.36±0.01	1.25±0.01	1.207±0.016
Diclofenac (10 mg/kg)	00.75±0.04** (18 %)	0.77±0.33** (19 %)	0.88±0.05** (18 %)	0.86±0.02** (37 %)	0.78±0.03** (38 %)	0.77±0.03** (37 %)
ME (200 mg/kg)	0.88±0.03 (3.3 %)	0.90±0.03 (5.3 %)	0.93±0.03** (13 %)	0.94±0.05** (31 %)	0.92±0.04** (26 %)	0.90±0.05** (25 %)

Values are expressed as mean±SEM (standard error of the mean) of 6 determinations.

** *p*<0.01, compared to normal

The increase in dry weight of cotton pellet granuloma was compared and it was found that the alcohol extract of ME inhibited the increase in dry weight by 29% as compared to saline control while diclofenac showed 45% inhibition as compared to control (Table 2). The alcohol extract of ME significantly reduced acetic acid-induced writhings and strechings in mice as compared to control with a 34% reduction as compared to normal and 52% reduction in writhing was seen in diclofenac-treated mice (Table 3). Brewer’s yeast at a dose of 20 mg/kg s.c. increased the rectal temperature by about 2°. Alcohol extract of ME reduced the elevated rectal temperature up to 5 h following its administration. The antipyretic activity started at 1 h and the effect was maintained for 5 h. The response was comparable to that of paracetamol (Table 4). Alcohol extract of ME did not reduce the rectal temperature in normal rats.

**TABLE 2:** EFFECT OF ALCOHOLIC EXTRACT OF MIMUSOPS ELENGI ON COTTON PELLET INDUCED GRANULOMA IN RATS

Treatment	Wet weight (mg)	Dry weight (mg)	% of inhibition	Transudative Weight (mg)	% of inhibition
Control	137.8±8.13	58.17±4.84		79.67±8.90	
Diclofenac (10 mg/kg)	75.83±4.25**	32.5±2.32**	44.2	43.33±2.84**	45.7
ME (200 mg/kg)	94.33±3.62**	41.33±4.33	29	53±2.35**	33.5

Values are expressed as mean±SEM (standard error of the mean) of 6 determinations.

** *p*<0.01, compared to normal

**TABLE 3:** EFFECT OF ALCOHOLIC EXTRACT OF MIMUSOPS ELENGI ON ACETIC ACID INDUCED WRITHING IN MICE

Treatment	Number of writhing in 15 min	Percentage of inhibition
Normal saline	94.00±5.17	
Diclofenac (10 mg/kg)	45.33±4.60**	51.78
ME (200 mg/kg)	62.17±4.56**	33.87

Values are expressed as mean±SEM (standard error of the mean) of 6 determinations.

** *p*<0.01, compared to normal

**TABLE 4:** EFFECT OF ALCOHOLIC EXTRACT OF MIMUSOPS ELENGI ON BREWER’S YEAST INDUCED PYREXIA IN RATS

Treatment	Rectal temperature (°)							
	Before yeast	18 h after	Temperature after treatment					
			30 min	1 h	2 h	3 h	4 h	5 h
Normal Saline	36.47±0.2	38.15±0.3	38.18±0.2	38.23±0.1	38.37±0.1	38.48±0.1	38.62±0.1	38.63±0.1
Paracetamol (150 mg/ kg)	36.25±0.1	38.55±0.2	37.68±0.1	37.40±0.1^b^	37.07±0.1^b^	36.68±0.1^b^	36.50±0.1^b^	36.63±0.2^b^
ME (200 mg/kg)	36.42±0.2	38.38±0.3	38.00±0.1	37.73±0.1	37.53±0.2^b^	37.35±0.1^b^	37.52±0.3^b^	37.78±0.2^b^

Values are expressed as mean±SEM (standard error of the mean) of 6 determinations. b=*p*<0.01, compared to normal.

The present study revealed that alcohol extract of ME possessed significant antiinflammatory and analgesic activities in experimental animals at a dose of 200 mg/kg. The antiinflammatory activity of alcohol extract of ME was evaluated using acute (carrageenan) and sub acute (cotton pellet) models of inflammation. Carrageenan-induced paw oedema as an *in vivo* model of inflammation was selected to assess the antiinflammatory activity of natural product particularly in the acute phase of inflammation[20]. Oedema formation due to carrageenan in the rat is a biphasic event. The early phase is related to the production of histamine, serotonin and possibly cyclooxygenase products and kinin like substances reaching peak at 180 min. The second phase of oedema is due to the release of prostaglandins, free radicals, proteases and lysosomes. The second phase is sensitive to most clinically effective antiinflammatory drugs[21,22]. Oral administration of the alcohol extract of ME suppressed the edematous response after 2 h and this effect continued upto 5 h. The observed effect was similar to that of diclofenac. NSAIDS block the synthesis of prostaglandins by inhibiting cyclooxygenase. Naturally occurring polyphenols such as flavonoids, coumarins and saponins might be expected to interfere with the process of synthesis of prostaglandins to produce antiinflammatory effects[23,24]. The results of the present study indicated that alcoholic extract of ME significantly inhibited the formation of rat hind paw oedema in the late phase. The effect of ME may be due to influence on inflammatory mediators and also on pathway of prostaglandins synthesis which may be due to the presence of flavonoids, saponins and other polyphenolic compounds in ME. The cotton pellet granuloma method has been widely used to assess the transudative, exudative and proliferative phase of sub acute inflammation. The inflammatory granuloma is a typical feature of the subacute inflammatory reaction. The fluid absorbed by the pellet greatly influences the wet weight of the granuloma whereas the dry weight correlates well with the amount of granulomatous tissue formed[25]. Based on the results presented in Table 2, it can be concluded that the alcohol extract of ME significantly inhibits the wet weight of granuloma suggesting that the alcohol extract of ME inhibited vascular permeability. When assessment was made on the dry weight of granuloma, the alcohol extract of ME found to inhibit granuloma formation also indicating the effect of alcohol extract on the proliferative phase of inflammation.

Two different analgesic testing models were employed with the objective of identifying peripheral (acetic acid-induced writhing method) and central analgesic effect (hot plate and tail clip method) of the test substances. The acetic acid-induced writhing is a visceral pain model that has been associated with release of arachidonic acid, cyclooxygenase, bradykinins and substance P. Prostaglandin (PGE_2_and PGF_2α_) biosynthesis plays a role in nociceptive mechanisms[26]. From the results obtained (Table 3) the alcohol extract of ME showed analgesic effect in acetic acid-induced writhing probably by inhibiting prostaglandin synthesis. In our present study ME failed to exhibit any significant analgesic activity in the hot plate and the tail clip models of analgesia which reveals that the analgesic activity of alcohol extract of ME is of the type produced by non-narcotic analgesics.

In the antipyretic test, alcoholic extract of ME markedly decreased elevated body temperature but not in normal animals. Yeast-induced fever is called pathogenic fever. Its etiology includes production of prostaglandins, particularly PGE _2_appears to be a final pathway responsible for fever production induced by several pyrogens[27]. Most of the NSAIDS show the antipyretic activity by inhibiting the prostaglandin synthesis. It is therefore suggested that the antipyretic effect of alcoholic extract of ME occurs in a similar fashion as paracetamol. In conclusion alcoholic extract of *Mimusops elengi* shows antiinflammatory, analgesic and antipyretic activities in rats. Further studies are in progress to isolate and identify the compounds which are responsible for these activities.
